# *In vitro* Comparison of Anti-microbial and Anti-adherence Activities of Three New Mineral Trioxide Aggregate Formulations against Species Associated with Failed Endodontic Treatment 

**DOI:** 10.30476/dentjods.2025.103996.2500

**Published:** 2025-12-01

**Authors:** Aida Mehdipour, Roohollah Fateh, Zeinab Naseri, Mohammad Aghaali, Ali Saleh, Faezeh Kabiri, Alireza Rasouli

**Affiliations:** 1 Cellular and Molecular Research Center, Qom University of Medical Sciences, Qom, Iran.; 2 Student Research Committee, Qom University of Medical Sciences, Qom, Iran.; 3 Dept. of Community Medicine, School of Medicine, Qom University of Medical Sciences, Qom, Iran.; 4 Dept. of Microbiology, Faculty of Basic Sciences, Qom Branch, Islamic Azad University, Qom, Iran.

**Keywords:** Endodontic, Mineral trioxide aggregate, *Enterococcus faecalis*, *Candida albicans*

## Abstract

**Background:**

Root canal treatment is one of the most critical dental treatments that help to maintain teeth. However, the growth of microorganisms leads to treatment failure. Considering the widespread use of mineral trioxide aggregate (MTA) in endodontic treatment, *in vitro* comparison of antibacterial, antifungal and anti-adherence activities of new types of MTA materials is indispensable.

**Purpose:**

This study aimed to investigate and compare the antibacterial, antifungal, and anti-adhesion properties of three types of MTA, including A.G.M MTA (Andishe Gostar Masud, Tehran, Iran), Ortho MTA (BioMTA, South Korea), and Ultradent™ MTA Flow (Ultradent MTA Flow, USA).

**Materials and Method:**

In this laboratory study, the antibacterial activity of three MTA substances against the strain of *Enterococcus faecalis* (*E. faecalis*) was carried out by the modified direct contact test (MDCT) method in 1, 3, 7, and 14 days. Antifungal activity of MTA against *Candida albicans* (*C. albicans*) was performed using a tube-dilution test in 1, 24, and 72 hours. The antibiofilm property of three MTA substances against *E. faecalis* strain was determined using the crystal violet staining method and measurement by ELISA.

**Results:**

Ortho MTA, A.G.M MTA, and MTA Flow showed intense antibacterial activity. The difference in the antibacterial effect of the three types of MTA was not statistically significant in all periods (*p* Value> 0.05). In the investigation of antifungal properties after 72 hours, all three types of MTA had antifungal properties (*p*< 0.050). Comparing the anti-adhesion properties of three MTA substances on *E. faecalis*, it is not significant (*p*< 0.05).

**Conclusion:**

A.G.M MTA showed the highest antibacterial activity, and Ultradent MTA Flow and Ortho MTA showed stronger antifungal activity than A.G.M MTA. The highest anti-bacterial property was related to A.G.M MTA.

## Introduction

Endodontic treatment involves the complete removal of pulp tissue and is usually the only available option (besides tooth extraction) for patients with irreversible pulpitis [ 1 ]. The primary role in root canal treatment failure and exacerbation of periapical diseases is related to the presence of microorganisms. Therefore, eradicating root canal system microorganisms in root canal treatment and preventing the entry of bacteria into the root canal system is one of the main factors of treatment success [ 2 ]. The main challenge in root treatments is the removal of biofilms from the root system, so an additional challenge for the materials used is their antibacterial activity [ 3 ]. Most root canal treatment failures result from insufficient cleaning of the root canal and the exit of bacteria into the radicular space [ 4 ]. The prevalence of root canal treatment failure is about 6% [ 5 ]. Mineral trioxide aggregate (MTA) was introduced in dentistry in 1993 for the first time and received food and drug administration (FDA) approval in 1998. MTA has revolutionized dentistry and has become one of the most studied endodontic materials over the past two decades [ 6 ]. MTA has antifungal and antibacterial properties due to its liquid-to-powder ratio and is composed of bismuth, silica, and calcium [ 7 ]. Bismuth lipophilic nanoparticles (BisBAL NPs) provide antimicrobial and antibiofilm activity to MTA [ 8 ]. Today, MTA is used for the treatment of pulp cap, pulpotomy, and apexogenesis, creating an apical barrier in necrotic teeth with an open apex, repairing root perforations, treating root resorption, treating teeth with mid-root fractures, and also canal filling material. The materials used for apexification, perforation repair, or retrograde root filling are more challenging compared to the material used for conventional root canal filling, as they have a more contact surface with the underlying periodontal tissues. MTA has favorable properties such as antimicrobial and antifungal properties, a lack of solubility in tissue fluid, biocompatibility, stimulation of hard tissue formation, and high pH. The most important properties of MTA in dentistry are its biocompatibility and flooding ability. The obtained flood, due to its ability to contract and expand, which is similar to dentin, leads to high resistance to bacterial migration and microleakage into the root canal system. Creating a stable barrier against bacterial leakage is one of the critical factors of clinical success [ 5 , 9 - 10 ].

Contradictory results about the antimicrobial properties of MTA have been obtained in previous studies. Torabinejad *et al*. [ 11 ] stated that MTA was not effective on *Bacillus subtilis* (*B. subtilis*), *Staphylococcus aureus* (*S. aureus*), and *Enterococcus faecalis* (*E. faecalis*). Still, it was also found that the antibacterial effect against *Streptococcus mitis* (*S. mitis*) was more significant with fresh MTA. Bhavana *et al*. [ 4 ] concluded that the antimicrobial activity of Biodentine against* E. faecalis* and *Candida albicans* (*C. albicans*) is higher than that of MTA. Esteki *et al*. [ 12 ] showed that MTA has antimicrobial effects against *E. faecalis* and *C. albicans*.

MTA Flow (Ultradent MTA Flow, USA) is composed of powder and gel. Bismuth oxide, tricalcium silicate, dicalcium powder, and water-soluble silicone liquid gel are the ingredients of this material. The characteristics of MTA Flow include high radio-opacity, low solubility, the ability to form calcium phosphate deposits and a certain alkaline activity on its surface. It was recently reported that MTA Flow, compared to ProRoot MTA, is biocompatible and less cytotoxic [ 13 ]. Ortho MTA (BioMTA, South Korea) is made to achieve maximum benefits and clinical performance. According to previous study, Ortho MTA showed antibacterial properties, high biocompatibility, and less microleakage [ 14 ]. Microbes commonly found in the root canals of teeth with endodontic disease after endodontic treatment are mainly gram-positive, such as *Actinomyces* and *Propionibacterium*, and cocci, such as *Enterococcus* and *Streptococcus* [ 15 ].

C. albicans is often isolated from root infections. Although it is recognized by the dental pulp and surrounding radicular tissue cells that mount immune responses, it escapes the host's defenses and causes cell death. Then,* C. albicans* attaches to the dentin, forms a biofilm, and invades the dentinal tubules to resist intracanal disinfectants and endodontic treatments. This fungus is insensitive to most common materials and survives inside biofilms and dentinal tubules [ 16 ].* E. faecalis* is the primary etiologic agent of periradicular tissue lesions that develop following root canal treatment. According to Esteki *et al*. [ 12 ] study, this microorganism was isolated from 22 to 77% of teeth with failed endodontic treatments.* E. faecalis* is resistant to high pH and can attack dentinal tubules. Therefore, it is very resistant to intra-canal materials.* E. faecalis* is the predominant microorganism in chronic apical periodontitis. Compared to other root microorganisms, it is more resistant to local antiseptic agents [ 17 ]. 

Evaluation according to clinical criteria shows that complete removal of infected dentin does not eliminate bacteria and the prevention and treatment of pulp inflammation; using substances with high antibacterial properties is necessary [ 18 ]. Bacterial adhesion is essential in tissue colonization and depends on the bacterial cell surface properties [ 19 ]. The findings suggest that interfering with bacterial adhesion could be a new tool to fight infectious diseases. Anti-adhesion-based therapies can effectively prevent and treat bacterial infections [ 20 ].

A.G.M MTA (A.G.M Dental MTA cement; Andishe Gostar Masud, Tehran, Iran) is a bioceramic endo cement and is a new product in the Iranian market, but it has not been investigated and studied. The antibacterial and antifungal properties of three types of MTA available in Iranian markets were examined in the present study. Given the limited research on the antibacterial, antifungal, and anti-adhesion properties of A.G.M MTA, Ortho MTA, and Ultradent Flow MTA, this study aimed to compare these properties to gain a better understanding of their effectiveness in treating root canal infections.

## Materials and Method

This study was approved by the Human Ethics Committee of Qom University of Medical Sciences (IR.MUQ. REC.1402.101), Qom, Iran. The present research was an *in vitro* study in 2023-2024. The statistical population under study comprised mixed mineral three-oxide bio-ceramics with the appropriate consistency of the mentioned brands. This laboratory study investigated the antibacterial and anti-adhesion activity of various bioceramic materials against *E.faecalis* (ATTC 29212) and their antifungal activity against *C.albicans* (ATTC 10231).

### Antibacterial properties of bioceramics

**Figure 1:**
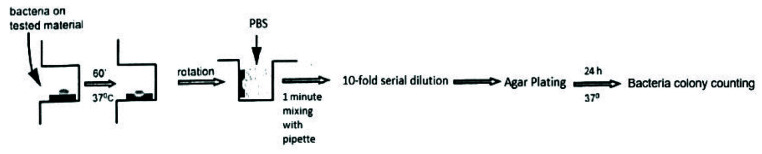
Image of modified direct contact test (MDCT) method

The standard suspension of* E. faecalis* was cultured in brain heart infusion (BHI) agar medium for 18 hours at 37°C. Then, the bacterial suspensions were centrifuged at 5000 rpm for 5 minutes at room temperature, and 0.5 McFarland concentration was prepared from the bacterial strain in phosphate-buffered saline (PBS). Finally, the antibacterial activity of Ortho MTA, A.G.M MTA, and MTA Flow against* E. faecalis* was investigated by the modified direct contact test (MDCT) method (Figure 1). First, three types of MTA were prepared according to the manufacturer's instructions, and every kind of MTA was placed vertically on the side wall of the 96-well microtiter plate using a dental tool with a round end (about a quarter of the well was filled). Five plates (96-well), containing MTA (corresponding to 5 time periods), were prepared and freshly mixed for 20 minutes, 24 hours, 3 days, 7 days and 14 days in 100% humidity at 37°C. At the beginning of each period, 10μL of bacterial suspension (approximately 2×10^6^ CFU/ mL) was carefully placed on the surface of each MTA. 10μL of bacterial suspension as a control group was placed on one of the side walls of the empty wells of the plates. After incubation of the samples, to evaporate the liquid suspension, the plate was placed in a vertical position for 1 hour at 37°C. After that, 300μL of PBS was added to all 4 wells (3 wells containing 3 different types of MTA+1 well containing the control group). After slow mixing with a pipette for 1 min, 10-fold serial dilutions were made using PBS. After completing the serial dilution process from each microtube (10 microtubes corresponding to 10 different dilutions), 100μL of solution was transferred to the corresponding agar plates. In general, 20 agar plates were used in each period, so five plates were considered for each of the three types of MTA along with the control group, each plate containing two different dilutions. The plates were incubated at 37°C for 24 hours to measure the survival of the bacteria. Then, 24 hours after incubation, CFU/ml was measured by counting colonies on the agar plate. Experiments were performed in triplicate [ 21 ]. 

### Antifungal properties of bioceramics

First, three loops of* C. albicans* colonies (loop diameter 4mm) were added to 10 ml of Sabouraud infusion broth (SIB) and were incubated at 37°C for 7 days. Then, 0.5 grams of powder of each type of MTA was prepared, and the necessary amount of liquid was added to it according to the manufacturer's instructions. Each one was placed in one of the wells of the 12-well plate. After 24 hours, 1ml of Candida suspension was poured on MTA. Also, 500 μl of SIB and 500 μl of one-week* C. albicans* suspension were considered as positive controls. 1000μl of SIB was considered a negative control. Then, the plate was incubated at 37°C. After 1 hour, 1 day, and 3 days of incubation, at the end of each period, 50 μl were removed from each well and transferred to 2.5 ml of SIB in the corresponding laboratory tube. All tubes were incubated at 37°C. The growth was monitored for 7 consecutive days. The growth of fungi was checked daily for 7 days, based on the presence or absence of optical density (OD). Experiments were performed in triplicate [ 22 ].

### Anti-adhesion property of bioceramic materials against* E. faecalis*

The crystal violet staining method was used to check the anti-adhesion property.* E. faecalis* was freshly cultured at 37°C. MTAs were prepared as discs with a diameter of 5 mm and a thickness of 2mm and were kept in humid conditions at 37°C for 24 hours. The disks were placed in the bottom of the wells of a 12-well plate. The discs were sterilized with UV rays, and 3 ml of bacterial suspension and culture BHI-broth medium containing 5×10^6^ bacterial cells were added to the discs until the MTA surface was completely covered. The plate was incubated at 37°C. After 24 hours, the discs were washed twice with sterile water and then placed at 65°C for 15 minutes to fix. Then, staining with 0.3% crystal violet was done for 15 minutes. Several washes were performed with sterile water, and the samples were air-dried. Finally, to wash and separate the crystal violet attached to the biofilm, 96% ethanol was added to all discs. Then, 250 µL of the ethanol and disc mixture was transferred to the wells of a 96-well plate. OD at 630 nm to quantify crystal violet was read by Eliza Reeder (Biotech, Winooski, VT, USA). Experiments were performed in triplicate [ 23 ]. 

### Statistical Analysis

Results were expressed as means±standard error (SE) of three independent repeated experiments. Data were statistically analyzed with the Kruskal-Wallis and one-way ANOVA test using SPSS software version 20 soft-ware. 

## Results

**Figure 2:**
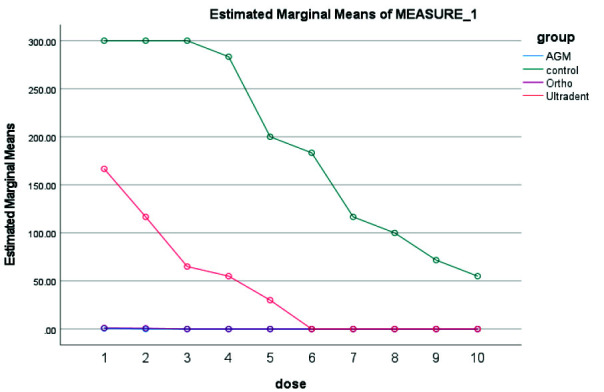
Results of antibacterial properties of three types of mineral trioxide aggregate (MTA) in 20 minutes in 10 serial dilutions. AGM mineral trioxide aggregate (A.G.M), Ortho mineral trioxide aggregate (Ortho), Ultradent™ MTA Flow (Ultradent)

The Kruskal Wallis test was used to check the antibacterial properties of MTA types. 10-fold serial dilutions using PBS were used in the 20 minutes, 24 hours, 3 days, 7 days, and 14 days for the three kinds of MTA studied. Ortho MTA initially showed a strong antibacterial effect but lost its antibacterial ability after 14 days. A.G.M MTA showed antibacterial solid activity from the beginning to the end of the first week. However, it showed little bacterial growth after 14 days. MTA Flow showed an antibacterial effect after 24 hours. The difference in the antibacterial effect of three types of MTA over time was not statistically significant. In the 20 minutes, A.G.M MTA showed the highest and Ultradent MTA the lowest antibacterial properties (Figure 2).

**Figure 3:**
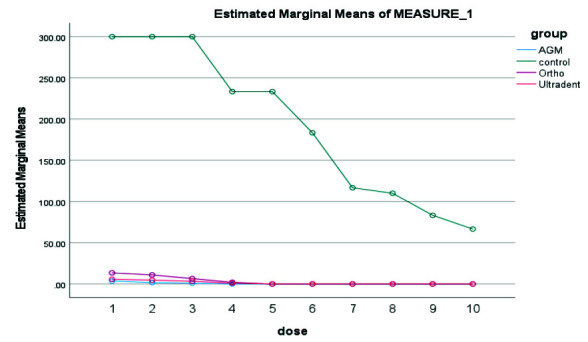
Results of antibacterial properties of three types of mineral trioxide aggregate (MTA) in 24 hours in 10 serial dilutions. AGM mineral trioxide aggregate (A.G.M), Ortho mineral trioxide aggregate (Ortho), Ultradent™ MTA Flow (Ultradent)

**Figure 4:**
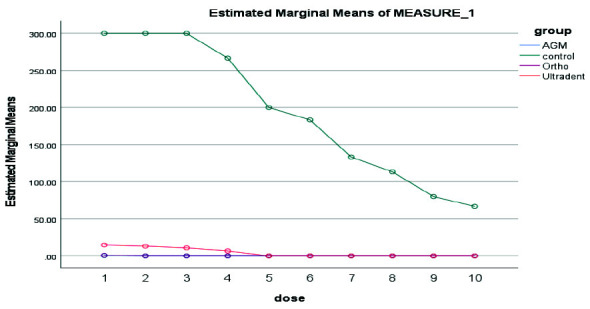
Results of antibacterial properties of three types of mineral trioxide aggregate (MTA) during 3 days in 10 serial dilutions. AGM mineral trioxide aggregate (A.G.M), Ortho mineral trioxide aggregate (Ortho), Ultradent™ MTA Flow (Ultradent)

**Figure 5:**
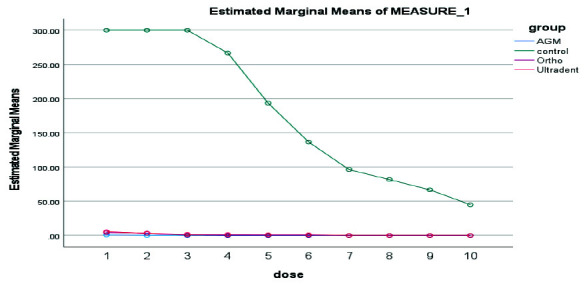
Results of antibacterial properties of three types of mineral trioxide aggregate (MTA) during 7 days in 10 serial dilutions. AGM mineral trioxide aggregate (A.G.M), Ortho mineral trioxide aggregate (Ortho), Ultradent™ MTA Flow (Ultradent)

**Figure 6:**
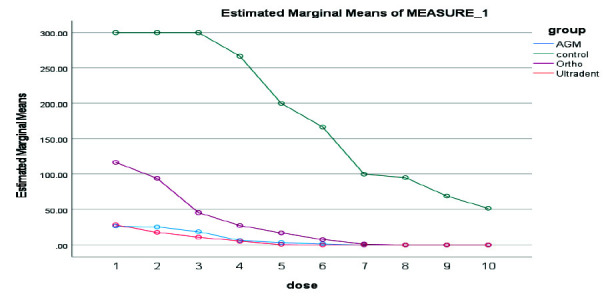
Results of antibacterial properties of three types of MTA during 14 days in 10 serial dilutions, AGM mineral trioxide aggregate (A.G.M), Ortho mineral trioxide aggregate (Ortho), Ultradent™ MTA Flow (Ultradent)

**Figure 7:**
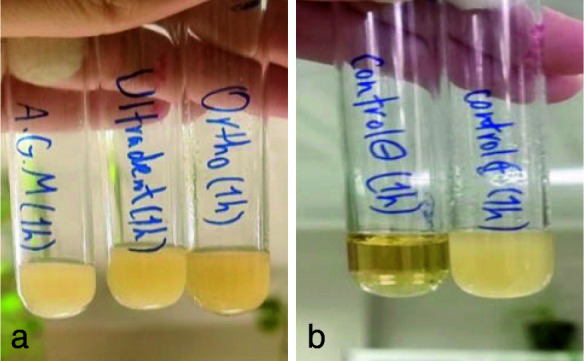
**a:** Fungal growth in all three types of mineral trioxide aggregate (MTA) (1 hour group), **b:** Positive control on the right side and negative control on the left side (1-hour group)

**Figure 8:**
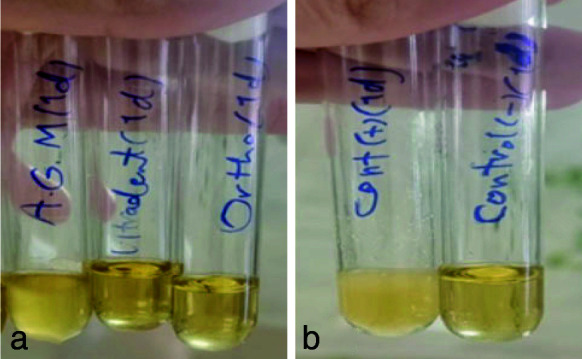
**a:** Fungal growth was observed only in A.G.M mineral trioxide aggregate (MTA) (one-day group), **b:** negative control on the right and positive control on the left (one-day group)

**Figure 9:**
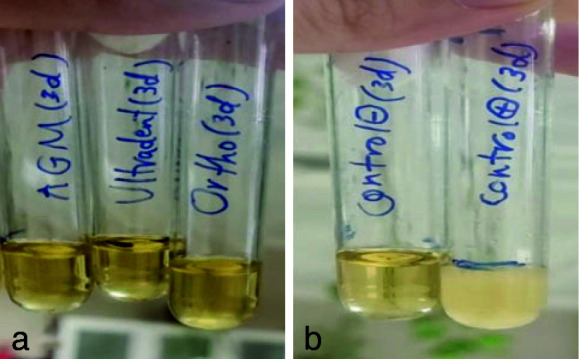
**a:** Absence of turbidity and lack of fungal growth in all three types of mineral trioxide aggregate (MTA) in (3-day group), **b:** Positive control on the right side and negative control on the left side (3-day group)

In the 24 hours, A.G.M MTA showed the highest and Ultradent MTA the lowest antibacterial properties (Figure 3). In 3 days, A.G.M MTA showed the highest and Ultradent MTA the lowest antibacterial properties (Figure 4). In 7 days, A.G.M MTA showed the highest, and Ultradent MTA had the lowest antibacterial properties (Figure 5). In 14 days, A.G.M MTA showed the highest and Ortho MTA the lowest antibacterial properties (Figure 6). The Kruskal Wallis test was used to check the antifungal properties of MTA types. In the present study, the results showed that the growth of fungi in all three types of MTA decreased over time. In the 1-hour test, all three classes of MTA could not inhibit fungal growth (Figure 7). Ortho MTA and MTA Flow inhibited fungal growth within 24 hours (Figure 8). In the 72-hour study, all three types of MTA had antifungal properties (Figure 9). 

In the present study, the descriptive data related to the anti-bacterial properties of the three types of MTA studied are presented in Table 1. A.G.M MTA has the highest anti-bacterial effect (lowest OD number), and Ortho MTA and Flow MTA are ranked second and third in terms of anti-bacterial properties. The difference between these three groups was not statistically significant (*p*= 0.410). 

**Table 1:** Descriptive data related to the anti-adhesive properties of three types of mineral trioxide aggregate (MTA) according to One-way ANOVA analysis

MTA	Average	Standard deviation	95% COI		Minimal	Maximal	*p* Value
			Minimum	Maximum			
A.G.M	0.65	0.06	0.48	0.81	0.58	0.71	0.410
Ortho	0.64	0.06	0.48	0.81	0.59	0.72	
Ultradent	0.71	0.06	0.54	0.88	0.65	0.79	
Total	0.67	0.06	0.62	0.72	0.58	0.79	-

A.G.M: AGM mineral trioxide aggregate; Ortho: Ortho mineral trioxide aggregate; Ultradent: Ultradent™ MTA Flow

95% COI (confidence interval for the mean)

## Discussion

The antibacterial properties of three types of MTA based on calcium silicate were investigated and compared in this investigation. Ortho MTA contains various ingredients including bismuth oxide, free calcium oxide, gypsum, tetracalcium aluminoferrite, tricalcium aluminate, dicalcium silicate, and tricalcium silicate [ 24 ]. MTA flow is a new formulation of MTA that can be used in different concentrations and there are very few studies on the antimicrobial activity of MTA Flow [ 25 - 26 ].

According to the manufacturer's instructions, MTA Flow contains tricalcium silicate and dicalcium silicate. A.G.M MTA contains different phases of calcium silicate, aluminum silicate, and zirconium oxide. This type of MTA is a new product in the Iranian market that was introduced in 2020, and it has not been investigated and studied so far.

According to the results of the present study, Ortho MTA lost its antibacterial ability after 14 days. A.G.M MTA showed relatively high antibacterial activity from the beginning to the end of the first week and was able to inhibit more bacterial growth than the other two MTAs; after 14 days, it showed almost the same antibacterial activity as MTA Flow. The calcium silicates composition of the A.G.M MTA should be able to release calcium and hydroxide ions by utilizing the moisture available in the root canal. Meanwhile, calcium hydroxide is also produced and reacts with phosphate to form water [ 27 ]. MTA Flow showed an antibacterial effect after 24 hours, and relatively high antibacterial activity was observed after 14 days. Miranda *et al*. [ 25 ], using agar diffusion test (ADT) and direct contact test (DCT) methods, reported that MTA Flow had no antimicrobial effect on* E. faecalis* after 24 and 48 hours in neither ADT nor DCT. This is the first study that evaluates the antibacterial activity of MTA Flow using the MDCT method. The outcome of the current research showed that newly mixed MTA Flow showed the weakest antibacterial effect among the investigated materials. By examining different time intervals in our study, it was found that the antibacterial power of MTA Flow improved after 24 hours. The findings of the current study did not match the findings of the Miranda *et al*. study [ 25 ]. The inconsistency of the results can be due to the difference in the methods used in the two studies. Also, the current study examined the antibacterial effect after 14 days, while Miranda *et al*.’s study evaluated the antibacterial effect only in 24 and 48 hours.

Abduljabbar *et al*. [ 28 ] investigated the antibacterial properties of several endodontic materials with bis calcium silicate for 7 days. The finding of this study showed that microbial inhibition by endodontic materials gradually increases from 24 hours to 7 days. The reason can be explained by stating that the antimicrobial properties of MTA are related to the release of calcium hydroxide ions produced from the hydration of tricalcium silicate [ 29 ]. That is, the pH value of freshly mixed MTA material is 10.2, which increases to 12.5 after 3 hours [ 30 ]. Therefore, this reason can justify the possible increase in the antibacterial properties of MTA Flow over time. For the moment, Ortho MTA lost its antibacterial effect after 14 days and showed the weakest antibacterial effect on the 14th day. The reasons for the decrease in antibacterial properties related to the passage of time in all three MTAs can be related to the decrease in the release of antibacterial components from the tested materials [ 31 ]. Meanwhile, A.G.M MTA had the strongest antibacterial activity among the three types of MTA, and in freshly mixed (fresh), 24-hour, 3-day, and 7-day intervals; it showed no growth or little growth of bacteria. However, not much bacterial growth was reported in this MTA after 14 days.

It has been shown that the local bactericidal effect of MTA sealers are achieved through an increase in pH due to the hydration cycle of calcium silicate [ 32 - 34 ]. Tricalcium silicate and dicalcium silicate hydrate, form an alkaline calcium silicate gel when mixed with water. Calcium hydroxide releases hydroxyl ions from this calcium silicate matrix and thus creates high alkalinity and inhibits microbial growth [ 35 - 36 ]. The differences in the antibacterial activities of the three types of BioMTA in this study are probably the result of differences in their structure and composition. However, further research is required to investigate factors such as moisture, aging time, and bacteria species on the antibacterial activity of EndoSeal MTA.

So far, there is no report on the antifungal activity of A.G.M MTA, Ortho MTA, and Ultradent MTA Flow, this is one of the strengths of the present study.

In the present study, all three types of MTA were incapable of inhibiting the growth of* C. albicans* after one hour of contact. At 24 hours, Ortho MTA and MTA Flow did not show any fungal growth, but A.G.M MTA was still unable to inhibit fungal growth. In all three types of MTA, antifungal properties and no fungal growth were reported in the 72-hour study.

Weckwerth *et al*. [ 37 ] conducted a study on the sensitivity of oral* C. albicans* strains to different pH levels and aqueous solution saturated with calcium hydroxide and found that* C. albicans* could be killed entirely after 48 hours of direct contact, inhibited with saturated calcium hydroxide aqueous solution. The results of the present study were consistent with the results of Weckwerth *et al*.'s study [ 37 ].

Waltimo *et al*. [ 38 ] conducted a study to investigate the sensitivity of edible Candida species in a saturated aqueous solution of calcium hydroxide. They realized that the sensitivity of* C. albicans* strains was relatively low during short-term exposure to calcium hydroxide. However, 99.9% of Candida strains were killed after 6 hours of incubation. The findings of the present study were consistent with Waltimo *et al*.'s study [ 38 ]. Barbosa *et al*. [ 39 ] reported that a saturated solution of calcium hydroxide effectively kills* C. albicans* after 3 minutes of incubation.

This current study explored the anti-adhesion properties of three types of MTA materials against* E. faecalis*. As mentioned, there are a few studies on the antibacterial and antifungal properties of the three types of MTA studied. Also, the anti-adhesion property (anti-biofilm) of these three types of MTA has not been reported previously.

In the present study, among the three investigated materials, A.G.M MTA showed the lowest OD number. Ortho MTA was ranked second by a slight margin, and finally, MTA Flow had the third-highest OD number. However, this difference was not statistically significant, and as a result, no significant difference was reported between the 3 types of materials in terms of anti-adhesion properties.

With the biofilm formation increasing on the surface of discs, the absorption of crystal violet dye increases by biofilms. As a result, in the decolorization stage, a large amount of colors is released into the well or plate by ethanol, which increases the OD in the ELISA reader device. Therefore, with the increased amount of biofilm, the light encounter with the crystal violet particles also increases, which leads to the increase of OD [ 40 ].

One of the reasons for the antibiofilm activity of cement based on tricalcium silicate, such as MTA, is the increase in the pH of the environment during the setting process of material. The pH of the environment increases due to the formation of calcium hydroxide during the MTA hydration reaction [ 41 ]. Surface properties that are changed by environmental interactions may inhibit bacterial adhesion and, thus, biofilm formation [ 42 ].

In the present study, MDCT method is repeatable and quantitative. Compared to DCT, this modified version has maintained its advantages and improved some of its disadvantages. For example, MDCT allows measuring the bactericidal effect instead of the bacteriostatic effect of the endodontic material under investigation [ 33 ], which is essential in the clinical practice of root canal therapy [ 21 ]. Another critical advantage of MDCT is that the nature of the root sealers does not easily influence the results obtained. Root sealers that set more slowly are more likely to affect the reading of the results. Unset sealers cloud the culture medium during mixing, affecting light absorption and, ultimately, the spectrophotometer reading [ 43 ]. In MDCT, results are collected through bacterial culture in an agar medium, and quantitative counting of remaining bacteria is also possible [ 21 ].

The antifungal test method used in this study was adjusting the tube concentration or the tube-dilution test. This method effectively evaluates the antifungal properties of any filling material or solution [ 22 ]. This method allows direct contact between fungal cells and MTA. Sabouraud agar is a common medium for the isolation of edible yeasts. The pH of the environment is acidic (t-ypically 5.6), which allows the growth of yeasts and fungi while inhibiting the growth of most bacteria [ 44 ].

In this study, the crystal violet staining method was used to evaluate the adhesion of bacteria. Staining with violet crystal is simple and fast, and from an industrial point of view, it is a direct measurement method [ 45 ].

After root canal treatment failure, microbial flora in the canals is limited to a small number of microbial species, mainly gram-positive bacteria. Facultative anaerobic bacteria, especially* E. faecalis*, were the most common microorganisms isolated from teeth with failed endodontic treatment, which can survive through chemical washing or re-infect the root canal through microleakage. One of the main factors contributing to the growth of this bacterial species in the root canal system is its ability to adhere to tooth surfaces, which leads to biofilm formation. In addition,* E. faecalis* is superior to other bacterial species due to its ability to bind to collagen inside dentinal tubules even in an unfavorable environment [ 46 ]. Fungi are sporadically found in primary root canal infections; fungi are more commonly found in canals with failed treatment [ 47 ]. The presence of* C. albicans* in the oral flora is considered to be one of the possible reasons for the failure of some root canal treatments [ 48 ].* C. albicans* has been found in some root canal treatments. This phenomenon may be explained by the invasive nature of this fungus or its resistance to some intracanal drugs, such as calcium hydroxide [ 47 ].

Among the limitations of the present study was that other microorganisms causing root canal treatment failure, including Actinomyces Israelis and Propionibacterium, were not investigated. Periods longer than 14 days were not tested. More sealers should be tested in the future, along with their other properties, such as biocompatibility, cytotoxicity, and so on. Further studies are needed to confirm the current findings. Although all three investigated root sealers showed a certain degree of antibacterial activity, none of them could show a consistent antibacterial effect against* E. faecalis*.

Another limitation of this study was that evaluating the 72 hours of MTA cannot draw definitive conclusions regarding antifungal effects. Therefore, it is suggested that the antifungal activity of MTA be investigated for more extended periods.

One of the limitations related to the investigation of anti-adhesion properties is that the effect of antibacterial activity and the surface anti-adhesion properties of the studied materials may interfere with each other simultaneously. Therefore, more extended storage periods should be considered in future research so that the effect of these two variables can be separated and the direct antibacterial activity of the studied material can be evaluated regardless of the effect of surface characteristics. One of the reasons for this is that the bactericidal effect of the substance may decrease over time. Considering that the root infection has a polymicrobial etiology, examining* E. faecalis* alone may not separately reflect the definitive reason for the failure of endodontic treatments [ 49 ]. In several studies, various effects of adding compounds to MTA have been measured to improve its performance [ 50 - 51 ]. 

## Conclusion

The present results in this study showed that A.G.M MTA had the highest antibacterial activity. Regarding antifungal properties, MTA Flow and Ortho MTA showed higher antifungal activity. However, no significant difference was reported between the three types of MTA in the anti-adhesion property. Therefore, despite the non-significance of the difference in the antibacterial properties between the three types of MTA in this study, from a clinical point of view, it is possible to use A.G.M MTA for patients with recurrent or persistent root infection, especially those who have a previous medical history of antibiotic resistance considered. However, more studies in this field seem necessary in the future.
